# Identification of Fungal Dynamics Associated With Black Locust Leaves Mineralization and Their Correlations With Physicochemical Factors

**DOI:** 10.3389/fmicb.2020.00348

**Published:** 2020-04-07

**Authors:** Sihui Chen, Jing Zhang, Zhongming Wen

**Affiliations:** ^1^College of Grassland Agriculture, Northwest A&F University, Yangling, China; ^2^Shaanxi Provincial Land Engineering Construction Group Co., Ltd., Xi’an, China; ^3^Institute of Land Engineering and Technology, Shaanxi Provincial Land Engineering Construction Group Co., Ltd., Xi’an, China

**Keywords:** mineralization, fungal community, black locust, vegetation types, co-occurrence network

## Abstract

In this study, the fungal dynamics associated with black locust (BL) mineralization and its correlation with various environmental factors were evaluated across three different vegetation types along a gradient of temperature and humidity. The results confirmed that *Ascomycota* and *Basidiomycota* were the dominant phyla in each habitat, with average relative abundance of 86.57 and 11.42%, respectively. But both phylum abundance varied significantly among different BL leaves’ decomposing habitats. Black locust changed the most significantly in the forest habitat and the least in the steppe. In addition, the litter characteristics of BL decreased with total carbon and total nitrogen mineralization and underground water level in water-rich region, while this result was significantly consistent with the fungal diversity. Co-occurrence network studies revealed that significant correlations were found between fungal community composition and environmental factors, the decrease of underground water level influence the fungal structure in forest habitat. Finally, the present study results provide important insights about the biological invasion of new ecosystems.

## Introduction

The litter, or dead plant waste material accumulation on the upper surface of forests, is a common natural phenomenon and its mineralization by indigenous microbe play an important role in biogeochemical cycling and biodiversity (Manna et al., 2003; Vítková et al., 2015). However, over the last two decades, tropical forests have undergone fast deforestation for agricultural and economical purposes. Therefore, indigenous microbial dynamics have been influenced by intensive forest cutting and the native biological cycle has been seriously affected, which causes adverse impact on forest biodiversity (Otsuki et al., 2014). Mineralization is an important process at nature ecosystem (Addiscott, 2010). Plants obtain part of nutrients requirements through microbial mineralization from various organic sources (Liu et al., 2018). Plant nutrients could not take up from the soil and remain trapped in dead plant material and litter. Hence, the natural mineralization of deciduous forest litter through activities of different indigenous microbes is an important component for organic matter recycling and productivity (Eklind and Kirchmann, 2000; Zhao et al., 2019).

Fungi are important eukaryotic microorganisms that can survive in extreme environments (Robinson, 2010; Koivusaari et al., 2019). In terrestrial ecosystems, fungi are decomposers and parasites that affect total carbon, nitrogen cycling, and plant nutrition (Callaway et al., 2004; Heikki and Mary, 2004; Jain et al., 2019; Sarsaiya et al., 2019b). Microbial composition can be shaped by vegetation type (Hui et al., 2017). When new neighbors (plants) enter natural ecosystems, generally only a limited number of taxa succeed and propagate (Ludsin and Wolfe, 2001; Manna et al., 2003). Fungi participate in this process through the decomposition of complex organic substrates, with the different substrates affecting fungal composition in turn. Fungi are sensitive to environmental change, and environmental factors are the best predictors of fungal diversity and community composition at a large scale (Sarsaiya et al., 2019a). Plant growth is also related to environmental factors (Messier et al., 2010). Thus, the study of fungi is an effective approach for understanding the effects of litter decomposition (Pfeiffer et al., 2013; Yang et al., 2017) and biological invasion processes (Zhao et al., 2019).

Black locust (BL) was introduced to China at the beginning of the twentieth century and has been widely planted as a pioneer species on the Loess Plateau (Tateno et al., 2007). In recent decades, however, increasingly research shown that the species also causes severe environmental problems, including the overconsumption of underground water, quick turnover of composition and allelopathic potential of black locust (Vítková et al., 2015). The rapid spread and growth of its propagules allow its successful establishment over a wide range of environmental conditions (Vítková et al., 2015). For these reasons, it was listed as one of the most dangerous invasive species in Europe (Richardson et al., 2011; Benesperi et al., 2012). As a leguminous tree, BL participates in natural ecosystems, causing a decrease in native plant diversity (Rooney et al., 2010), sparse understory vegetation, and severe shifts in epiphytic lichen communities (Nascimbene and Marini, 2010; Richardson et al., 2011); it also impacts physicochemical characteristics, understory vegetation growth (Yüksek, 2012), and litter decomposition (Tateno et al., 2007). Physicochemical characteristics, such as nitrogen content and nitrogen availability, the structure of physicochemical characteristics, environmental quality, root biomass, and total organic carbon sequestration, can be improved greatly (Rice et al., 2004; Ussiri et al., 2006; Tateno et al., 2007; Yüksek, 2012), and the microbial diversity associated with litter decomposition has undergone dramatic changes (Manna et al., 2003; Qiu et al., 2010). However, most studies have focused on the bacterial community at specific sites, few on fungal community (Tao et al., 2018; Yang et al., 2018), and also regardless the influence of environmental heterogeneity. To illustrate the impact of BL in the native ecosystem, the responses of the fungal community, physicochemical characteristics and environment factors were selected.

The decomposition of BL leaf litter affects the local ecosystem, and the accumulation of its litter decreases the pH but increases the total carbon and nitrogen concentration (Vítková et al., 2015) and improves the ecological environment. However, the biogeochemical cycle is necessary to both environmental conditions and the rate of organic matter mineralization, which is closely related to the relative abundance (RA) of indigenous microbes. Microbial dynamics and their correlation to BL leaf litter are necessary to identify. Further field trials should also be performed that are technically executable, economically feasible, and most promising should then be applied by farmers for forest tree cultivation. The associated fungal diversity, physicochemical characteristics and their correlations with environmental factors are still unknown. Therefore, the objectives of this study are to (i) explore whether the decomposition of BL leaf litter impacts physicochemical characteristics and fungal community composition and diversity across environmental gradients, (ii) the cause of the change in the native ecosystem and (iii) undertake a reasonable evaluation of BL in semiarid regions.

## Materials and Methods

### Study Area

The study was carried out in the hilly-gully region of the Loess Plateau, China (36°23′–37°17′N, 108°45′–110°28′E). The climate is a semiarid continental climate. The average annual temperature was 8°C in the northwest (steppe habitat) to 10°C in the southeast (forest habitat). The precipitation varies from 420–539 mm (from steppe habitat in northwest to forest habitat in southeast), 70% of which falls in the period between June and September. The landscape is highly fragmented and characterized by hills and gullies; The climate and vegetation type in these three habitats (forest habitat in the south, forest-steppe habitat in the middle, and steppe habitat in the north) changes from southeast to northwest. As Loess plateau has suffered mostly from water erosion, many re-vegetation programs have been carried out in the region starting in the 1950s, including the “Grain for Green” project in 1999. BL has been planted extensively in the study area, including the steppe area. This provides an excellent opportunity to analyze the impact of the introduction of BL on the microbial community along an environmental gradient.

### Site Selection and Sample Collection

In the present study, our sampling sites were selected from three different vegetation habitats along a decreasing gradient of temperatures and humidity from northwest to southeast in Loess Plateau region (Otsuki et al., 2014). A total of 43 samples were collected from these three habitats to evaluate the effects of the decomposition of BL litter on microbial communities and physicochemical characteristics in comparison to those in the associated native plant community. FL (black locust in forest habitat, seven samples) and FN (native plant in forest habitat, eight samples) were collected from the forest habitat, EL (black locust in forest–steppe habitat, four samples) and EN (native plant in forest–steppe habitat, four samples) were collected from the forest–steppe habitat, and SL (black locust in steppe habitat, ten samples) and SN (native plant in steppe habitat, ten samples) were collected from the steppe habitat. The sampling points were 2,000 m apart from one another with similar slopes, aspects, and altitudes. This ensured that the replicates were true and reliable. At each 10 × 10 m sampling point, we collected five cores randomly located along an “S” line, which was established around the plants at a depth of 0–20 cm, and then we mixed the samples together. The mixtures were sieved through a 2 mm mesh to remove the roots, plant litter, and stones. Ten grams were placed into a 5 ml centrifuge tube and stored in an icebox during transportation and stored at −80°C until DNA extraction. The remaining samples were air-dried to analyze the physicochemical characteristics.

### Physicochemical Characteristics, Environmental Temperature, and Humidity

The pH was measured with a pH meter after shaking the water suspension for 30 min (room temperature = 23°C). The total organic carbon (TOC) was determined using the K_2_CrO_7_-H_2_SO_4_ oxidation method, and total nitrogen (N) was measured with the Kjeldahl method. Environmental temperature and humidity were recorded with iButtons (DS1923, Hygrochron, China) at 1.5 m above the ground (TA, HA), at the soil surface (TB and HB), and at 0–10 cm depth (TC and HC) every 30 min for 1 week.

### DNA Extraction, PCR, and High-Throughput Sequencing

DNA was extracted from the samples (500 mg wet weight) with the Fast-DNA< SPIN Kit (116560200 MP Biomedical, United States) according to the manufacturer’s instructions. The extracted DNA was diluted in TE buffer and stored at −20°C until use. ITS genes of distinct regions were amplified using specific primers (ITS1: 5′- CCGTAGGTGAACCTGCGG- 3′, ITS4: 5′- TCCTCCGCTTATTGATATGC - 3′) with the barcode (Bachy et al., 2013; Bengtsson-Palme et al., 2013). All reactions were performed in triplicate, and controls were included in each step. The amplicons were detected in a 1.5% (w/v) agarose gel. Sequencing was conducted on the Illumina-HiSeq 2500 platform at the Novogene Bioinformatics Technology Co., Ltd.

### Statistical Analysis

Analysis was performed using R platform (v3.2.2).^1^ Pairwise comparisons between means were conducted to analyze the effect of BL. NMDS (non-metric multi-dimensional scaling) of Brary–Curtis distance and CCA (canonical correspondence analysis) were used (to investigate the distribution of samples and relationship between groups and environmental factors, respectively vegan package in R). Kruskal–Wallis nonparametric testing (“agricolae” package) was used to distinguish significant differences among groups. Gephi was used for the network analysis, and the parameters (Liu et al., 2019) and genera with low abundances (<0.001%) were eliminated from each group (De et al., 2018). All correlations in the operational taxonomic unit (OTU) abundance were used to create a network in which each node represents one OTU, and each edge represents the correlation between the nodes and significant correlations between the nodes. Genera with the highest betweenness centrality values, which indicate the relevance of a node as capable of holding together communicating nodes, were considered keystone species (Vick-Majors et al., 2014). Methods in this study were provided in a flow diagram (Supplementary Figure S1).

## Results and Discussion

### Variation in Physicochemical Characteristics

The habitat, vegetation type, and physicochemical characteristics are summarized in Table 1. After the introduction of BL, total organic carbon and total nitrogen decreased with rapid growth of microbes and traits regardless of the living conditions, this was similar with the previous research (Yüksek, 2012). However, the results were inconsistent across the different habitats, and litter decomposition varied in the different habitats; the physicochemical characteristics on account of the decomposition of BL leaves changed the most in the forest habitats. The TOC and TN associated with BL were significantly lower than those associated with the native plants in the forest habitats and forest–steppe habitats, while the TOC and TN in FL were two times lower than those in FN, perhaps because of slow mineralization (Table 1). The present study results are consistent with previous researches (Tateno et al., 2007; Cui et al., 2018), which have reported that litter fall production and leaf litter decomposition between an exotic black locust plantation and indigenous oak forests were influenced by several physicochemical factors like moisture and aeration near Yanan on the Loess Plateau.

**TABLE 1:** Result of soil characteristics, pH value comparison between black locust and native plant, changed with vegetation habitats and types.

**Type**	**Soil organic carbon**	**Total nitrogen**	**Total phosphorus**	**pH value**
FL	11.27 ± 4.18bc	0.67 ± 0.19bc	0.59 ± 0.02a	7.02 ± 0.13a
FN	30.82 ± 15.65a	1.55 ± 0.69a	0.6 ± 0.03a	7.09 ± 0.22a
EL	8.6 ± 2.64c	0.53 ± 0.12c	0.6 ± 0.02ab	7.64 ± 0.06a
EN	19.05 ± 7.2ab	1.07 ± 0.39ab	0.56 ± 0.08a	7.46 ± 0.25a
SL	6.05 ± 1.58d	0.36 ± 0.09d	0.55 ± 0.01b	7.6 ± 0.1a
SN	5.96 ± 2.02d	0.33 ± 0.1d	0.55 ± 0.04b	7.57 ± 0.21a

*And the difference comparison among groups. Values within the same column followed by different letters indicate significant differences (*P* < 0.05, ANOVA, Kruskal–wallis test). Results are mean of replicates ± standard deviation. FL, black locust in forest habitat; FN, native plants in forest habitat; EL, black locust in forest-steppe habitat; EN, native plants in forest-steppe habitat; SL, black locust in steppe habitat; SN, native plants in steppe habitat.*

Some researchers have reported that physicochemical characteristics varied significantly within the different vegetation types and climate conditions (Qiu et al., 2010; Lazzaro et al., 2017). The Loess Plateau is located in a phosphorus-deficient region. Phosphorus changed more with habitat than with vegetation, and there was no significant difference after the litter decomposition of BL but a significant difference between the forest habitat and steppe habitat (Table 1 and Supplementary Table S1). The total phosphorus and pH values were stable after the introduction of BL, and the results were consistent across the three habitats (Table 1), this was similar with the previous research (Yang et al., 2017). While the BL in the different habitats also showed variation, the TOC, TN, and TP decreased from FL to SL (Table 1). As evident, BL tolerates various soil physicochemical properties (Vítková et al., 2015).

The pH value plays an important role not only in the activities, but also growth of microbes and related biogeochemical processes (Zhang et al., 2006; Nicol et al., 2010). According to previous research, pH can be affected by litter decomposition, vegetation type, and environmental factors (Tateno et al., 2007; Lazzaro et al., 2017). It was also found to affect plant species richness, for example there was an increase in richness from very acidic sites to somewhat less acidic sites (Schuster and Diekmann, 2003). The pH values associated with FL, EL, and SL were not significantly different, nor were those in the forest–steppe habitat and steppe habitat. Habitat was more important in determining the pH value than vegetation (Supplementary Table S1). This means that the ecosystem in the steppe was saline and hardened, which hampered the flourishing of plants, because the growth of BL was related to pH and water supply (Vítková et al., 2015). The physicochemical characteristics in the forest habitat, forest–steppe habitat, and steppe habitat indicated nutrient loss trends (Table 1). In the forest habitat, the vegetation seemed to be more abundant, and the microorganism communities developed under favorable conditions.

Similar to previous research, gradients of temperature and humidity were identified (Wang et al., 2015). From the forest habitat in the southeast to the steppe habitat in the northwest, the average temperature (TA) changed from 19.73 to 21.94°C, and the average humidity (HC) changed from 80.35 to 95.53% (Supplementary Table S2), the trends were similar with the previous research (Otsuki et al., 2014). When compared to the native vegetation communities, the introduction of BL caused changes in temperature, humidity, and physicochemical characteristics. The temperature (TA, TB, and TC) associated with FL was higher than that associated with FN, and similar trends were found for EL versus EN and SL versus SN, except for TC in SL versus SN (Supplementary Table S2). The temperature (TA, TB, and TC) associated with FL was higher than that associated with EL and SL. Humidity (HA, HB, and HC) significantly differed for EL versus EN. As a pioneer tree species, deciduous BL was first introduced to prevent water erosion and has been widely planted across the hilly-gully regions (Otsuki et al., 2014). The growth of BL is also strongly dependent on high water availability, there have been reports regarding BL deficient growth due to a lack of water (Motta et al., 2009). Comparisons of the humidity associated with the different vegetation types and habitats indicated that humidity tended to be influenced by vegetation type, which was significantly correlated with plant physiological traits. HC associated with FL was lower than that associated with FN (Supplementary Table S2). Black locust is an early successional plant in forests, and its rapid adaptability to various environments contributes greatly to its rampant colonization and spread (Manna et al., 2003; Vítková et al., 2015). Black locust grew quickly in a nutrient-rich region, following the overconsumption of water (Motta et al., 2009). In shrubland and grassland, the formation of an arbor layer affected the plant community, plant productivity, light regime, and microclimate (Kleinbauer et al., 2010); the fast growth of BL may contribute to increasing underground water and nutrition levels, and may significantly affect microbe growth.

### Distribution of Taxa and Fungal Community Diversity

**FIGURE 1:**
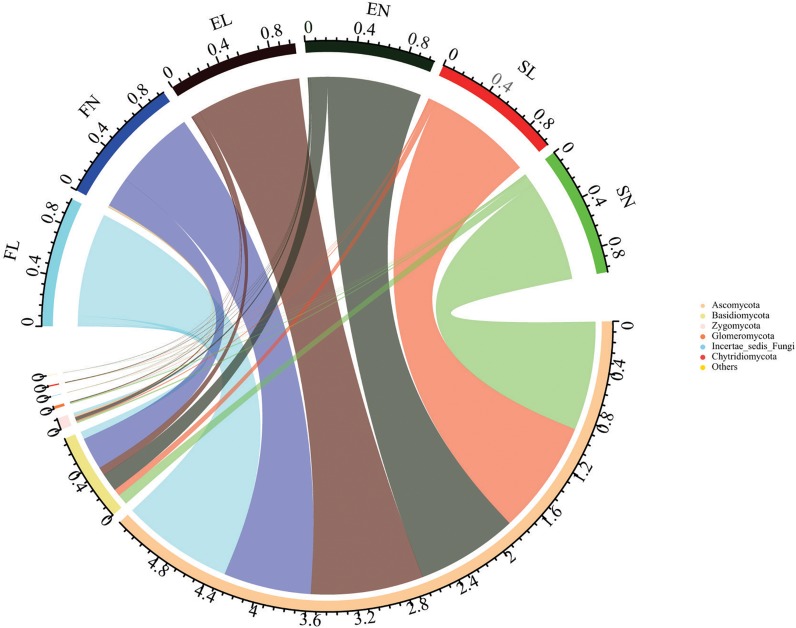
Composition of Fungal community at phylum level. The top six fungal phyla are shown, and the other phyla are included as “Others.” FL, black locust in forest habitat; FN, native plants in forest habitat; EL, black locust in forest-steppe habitat; EN, native plants in forest-steppe habitat; SL, black locust in steppe habitat; SN, native plants in steppe habitat.

A total of 3,632,154 effective sequence reads were obtained after removing low-quality reads and chimeras (Supplementary Table S3). The average number of sequences was 84,469 per sample (ranging from 63,582 to 91,687). The operational taxonomic unit (OTU) number was 34,718 at 97% similarity. The number of OTUs ranged from 479 to 1,111. Rarefaction curves (Supplementary Figure S2) tended toward saturation for almost every sample, indicating that the OTUs were representative of the total fungal community and that the fungal diversity index represented the variance of the different groups (Guo et al., 2019). In the fungal diversity analysis, the obtained sequences were classified at different taxonomic levels. The top six most abundant phyla are shown in Figure 1. Among the 43 samples from the three habitats, *Ascomycota* and *Basidiomycota* were the dominant phyla in each habitat, with average relative abundances of 86.57 and 11.42%, respectively. Each sample had similar content but different percentages at the phylum level. The proportions of *Ascomycota* and *Basidiomycota* varied among the different samples. The invasion of BL increased the relative abundance of *Ascomycota* and *Glomeromycota* in the forest habitat but decreased that of *Basidiomycota* in the forest habitat and *Glomeromycota* in the steppe habitat (Supplementary Figure S3). In the previous researches, the invasion plant usually effects the soil fungal diversity and the native ecosystem (Stefanowicz et al., 2017; Zhao et al., 2019). Unlike the physicochemical characteristics results, the invasion of BL changed the fungal community in the different habitats (Supplementary Table S4). Similar fungal dynamics with litter decomposition was also reported by Heikki and Mary (2004) and Tao et al. (2018) across a litter profile of the Chinese Loess Plateau and their responses to nitrogen inputs.

**FIGURE 2:**
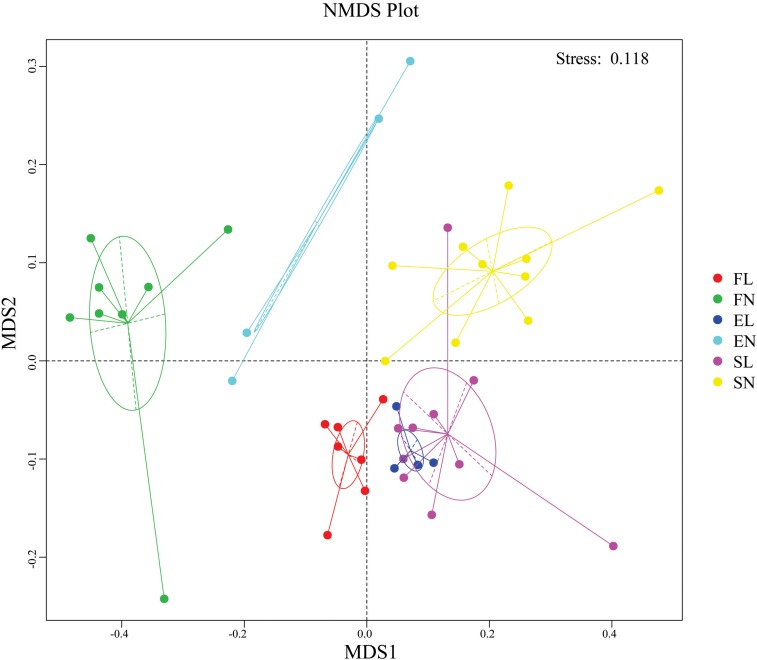
NMDS analysis were used to show the distribution of sample. NonMetric MultiDimensional Scaling, FL, black locust in forest habitat; FN, native plants in forest habitat; EL, black locust in forest-steppe habitat; EN, native plants in forest-steppe habitat; SL, black locust in steppe habitat; SN, native plants in steppe habitat.

**FIGURE 3:**
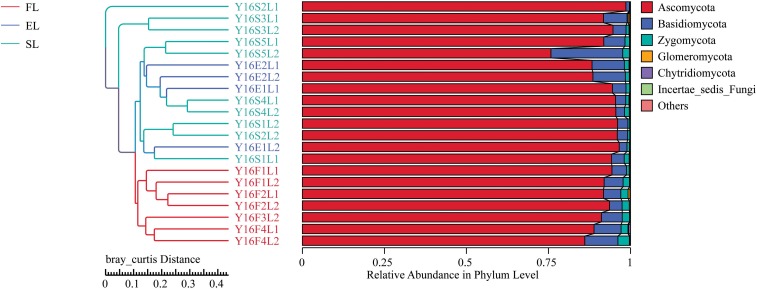
UPGMA poly-tree of black locust in different habitats. This indicates the relationship between different groups. Results are mean of replicates ± standard deviation. UPGMA, unweighted pair-group method with arithmetic means; FL, black locust in forest habitat; FN, native plants in forest habitat; EL, black locust in forest-steppe habitat; EN, native plants in forest-steppe habitat; SL, black locust in steppe habitat; SN, native plants in steppe habitat.

The NMDS results indicate that BL was well separated from native plants along axis NMDS2 and that axis NMDS1 explained the separation of FL and FN from SL and SN (Figure 2). In the α-diversity analysis, habitat had more pronounced and significant effects than vegetation type, and FL was significantly different from EL and SL (Table 2). Additionally, when testing the significance of community structure differences among groups, the forest samples of BL differed from the other samples. Based on the Bray–Curtis distance, the BL in the forest habitat was a unique branch in the UPGMA phylogenetic tree, and the three BL groups also showed diverse expression (Figure 3). Black locust grows in a wide range of living conditions, quickly adapts to the natural ecosystem, and is also affected by the surrounding environment (Cierjacks et al., 2013). Additionally, the humidity conditions were different, and the BL was severely dependent on the water conditions, which may cause a change in fungal dynamics in different habitats (Vítková et al., 2015). While, fungal diversity and structure were also related to the distribution of water variance, organic matter and productivity gradients (Hiiesalu et al., 2017).

**TABLE 2:** α-diversity of fungal communities in different habitats and vegetation type. All data are presented as mean ± standard deviation.

	**OS**	**Chao1**	**ACE**
FL	648.86 ± 52.06a	894.69 ± 96.85a	892.95 ± 79.18a
FN	687.25 ± 77.08a	909.53 ± 94.16a	928.56 ± 94.54a
EL	536.25 ± 36.88b	730.52 ± 67.45b	741.55 ± 98.41b
EN	694 ± 127.64a	934.05 ± 202.26a	916.26 ± 136.04a
SL	538.8 ± 90.33b	698.65 ± 119.39b	694.13 ± 131.71b
SN	573.2 ± 132.1ab	753.38 ± 173.82b	754.53 ± 169.06b

*FL, black locust in forest habitat; FN, native plants in forest habitat; EL, black locust in forest-steppe habitat; EN, native plants in forest-steppe habitat; SL, black locust in steppe habitat; SN, native plants in steppe habitat; OS, observed species; chao-index with chao1 algorithm to estimate the number of OTU in the community, ACE-index to estimate the number of OTU in the community. Results are mean of replicates ± standard deviation.*

### Relationship Between Fungi and Environmental Characteristics

**FIGURE 4:**
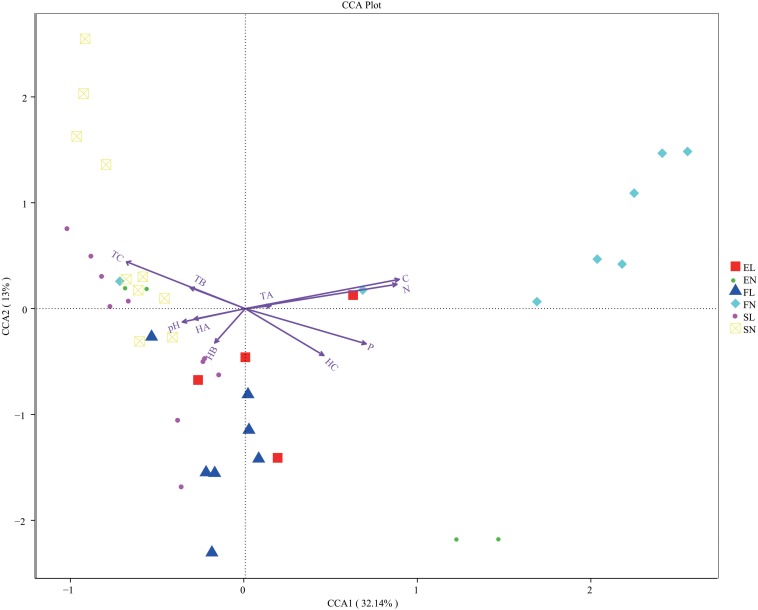
CCA analysis of three zones. The arrows represent different environmental factors, longer ray means greater the influence of the environmental factor. The angle between environmental factors represents the relationship between environmental factors and sample, acute means there is a positive correlation between the two environmental factors, while when it is obtuse, there is a negative correlation. CCA, canonical correspondence analysis; FL, black locust in forest habitat; FN, native plants in forest habitat; EL, black locust in forest-steppe habitat; EN, native plants in forest-steppe habitat; SL, black locust in steppe habitat; SN, native plants in steppe habitat. Results are mean of replicates ± standard deviation.

To determine the relative contribution of environmental factors to fungal structure and diversity, ten factors were taken into consideration, including total organic carbon, total nitrogen, total phosphorus, pH, temperature (TA, TB, and TC), and humidity (HA, HB, and HC). The CCA showed that the first and second CCA components explained 45.14% of the total variation in fungi according to the physicochemical characteristics (Figure 4). In the CCA, native plants were found to be different from the invasive plant (Figure 4), different limits for each group and this was consistent with the previous research (Lazzaro et al., 2017); this result was the same as those in the statistical analysis (Supplementary Table S1). In addition, there was a significant difference in physicochemical characteristics and environment factors in forest habitat and forest steppe habitat on account of BL leaf litter (Supplementary Table S5), and similar results were also found in recent research on invasive plants (Lazzaro et al., 2017). This indicates that the decomposition of BL leaf litter changed the native ecological system in the forest habitat, similar to what occurs in other plant invasions (Inderjit and van der Putten, 2010; Garbary et al., 2013).

The physicochemical characteristics, temperature, and humidity explained 64.15% of the total variation (Supplementary Figure S4), climate and environment condition were important for the fungal diversity variation (Peay et al., 2017). The forest habitat contains poplar and oaks and showed high values of physicochemical characteristics and humidity. Water- and nutrient-rich environments are essential for forest growth (Toledo et al., 2011; Kaspari et al., 2017). FL showed the opposite pattern, in line with BL growth traits, which quickly adapt to the local conditions, even acidic environments (Marschner, 1991; Vítková et al., 2015). EL was positively correlated with the surface temperature in the steppe habitat, but more parameters might be considered to explain the variance. EL was positively correlated with HC. In the steppe habitat, the relationship between samples and environmental factors was not clear. Some research has been reported that natural grassland had less relative carbon and phosphorus limitations than shrubland and forest(Cui et al., 2018). So more parameters might be taken into consideration for the sufficient explanation of the variance of steppe.

### Co-occurrence Network of Black Locust and Native Plants

**FIGURE 5:**
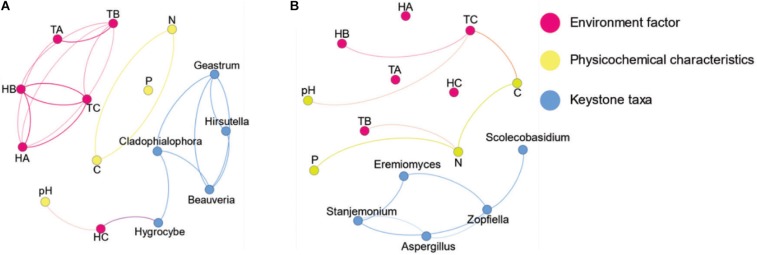
Co-occurrence networks of fungal communities in forest habitat and steppe habitat native plants without litter decomposition of black locust. **(A)** Black locust in the forest habitat and **(B)** native plants in the forest habitat. Blue node name-keystone nodes, red node name-environment factor (temperature and humidity), yellow node name-soil characteristics, FL, black locust in forest habitat; FN, native plants in forest habitat; EL, black locust in forest-steppe habitat; EN, native plants in forest-steppe habitat; HA, humidity at 1.5 m above the ground; HB, humidity at soil surface; HC, humidity at 0–10 cm depth of soil; SL, black locust in steppe habitat; SN, native plants in steppe habitat. TA, temperature at 1.5 m above the ground; TB, temperature at soil surface; TC, temperature at 0–10 cm depth of soil.

The co-occurrence network of fungi in the different groups of vegetation types and habitats (FL versus FN and SL versus SN) was constructed using network analysis based on significant correlations (Spearman’s correlation coefficients, *p* < 0.05). The resulting fungal network contained similar nodes for the same habitat, and the decomposition of BL litter decreased the number of links; there were two times the number of links for SN than SL, and SL had more links than FL (modularity index values >0.4). When the distribution of nodes was modularized, all nodes were grouped into several major modules (each module >10%). There were four major modules for FL, five for FN, and three for both SL and SN (Supplementary Figure S5). The networks for FN and SN were more complex than those for FL and SL (Supplementary Table S5). The top five genera identified as keystone taxa were *Cladophialophora*, *Geastrum*, *Hygrocybe*, *Beauveria*, and *Hirsutella* for FN and *Stanjemonium*, *Aspergillus*, *Eremiomyces*, *Scolecobasidium*, and *Zopfiella* for SN (Supplementary Table S6). For FN, *Hygrocybe* had a positive relationship with HC, while for SN, there were no keystone taxa with links to physicochemical characteristics, temperature, or humidity. Although the dominant taxa had more ecological niches and contained more energy in the ecosystem, keystone taxa can also function as drivers of the microbial community (Banerjee et al., 2018). In the co-occurrence network of FL, the decrease in underground humidity influenced the keystone taxa (Figure 5), which affected the structure and function of the microbial community.

Based on previous research of the relationship between keystone species and environmental factors (Liu et al., 2019), the introduction of BL decreases the forest habitat humidity and enhances the fungal community. As a fast-growing tree, BL also uses nutrients (Duan et al., 2017). Similar results were observed in the CCA, where the growth and development of FN were closely related to TOC and TN. The BL and native plant fungal community were most similar in the steppe habitat (Figure 2), and the physicochemical characteristics changed little. In the SN co-occurrence network, none of the environmental factors had a relationship with keystone taxa. The statistics showed that there was a significant difference between SL and SN (Supplementary Figure S2). More parameters are needed for the monitoring of BL in steppe habitat. Black locust is an important tree species on the Loess Plateau for forestry and soil erosion prevention. In an environment suitable for growth, the invasion of BL can promote soil water-holding capacity, nutrients levels, and fungal diversity. However, the invasion of BL will also lead to more severe problems, especially under water scarcity and in land that has been barren for a long time, resulting in more severe losses of soil quality, biodiversity, and water resources (Qiu et al., 2010).

## Conclusion

This study demonstrated that the effects of BL litter decomposition along with environmental gradients (temperature, humidity and vegetation type) were significantly influenced in physicochemical characteristics and their fungal community diversity. Black locust changed the most significantly in forest habitat and the least in steppe. The living conditions seemed more suitable for the trees in the forest and deteriorated from forest habitat to steppe habitat, while the vegetation type changed from trees to grassland. This study suggests that the assessment of the effects of the introducing of BL take the native vegetation type and local environment into consideration. Future work should consider keystone species of fungi based on our findings and the variance of underground water level.

## Data Availability Statement

The data generated for this study can be found in NCBI using the accession number PRJNA602415.

## Author Contributions

SC, JZ, and ZW conceived and designed this study. JZ participated in the sample collection and data analyses. SC completed the data analyses and the manuscript. ZW provided the constructive suggestions for revisions.

## Conflict of Interest

JZ was employed by the company Shaanxi Provincial Land Engineering Construction Group Co., Ltd. The remaining authors declare that the research was conducted in the absence of any commercial or financial relationships that could be construed as a potential conflict of interests. The reviewer YMD declared a shared affiliation, with no collaboration, with one of the authors, ZW, to the handling editor at the time of the review.

## Footnotes

1 http://www.r-project.org/

## Abbreviations

ACE-index: an index used to estimate the number of OTU in the community
BL: black locust
CCA: canonical correspondence analysis
chao1: index with chao1 algorithm to estimate the number of OTU in the community
EL: black locust in forest-steppe habitat
EN: native plants in forest-steppe habitat
FL: black locust in forest habitat
FN: native plants in forest habitat
HA: humidity at 1.5 m above the ground
HB: humidity at the soil surface
HC: humidity at 0–10 cm depth of soil
N: total nitrogen
NMDS: non-metric multi-dimensional scaling
OS: observed species
OTU: operational taxonomic unit
P: total phosphorus
RA: relative abundance
SL: black locust in steppe habitat
SN: native plants in steppe habitat
TA: temperature at 1.5 m above the ground
TB: temperature at the soil surface
TC: temperature at 0–10 cm depth of soil
TOC: total organic carbon
UPGMA: unweighted pair-group method with arithmetic means.

## References

[B1] Addiscott T. M. (2010). Soil mineralization: an emergent process? *Geoderma* 160 31–35. 10.1021/jf103508w 21190381

[B2] Bachy C. Dolan J. R. Lópezgarcía P. Deschamps P. Moreira D. (2013). Accuracy of protist diversity assessments: morphology compared with cloning and direct pyrosequencing of 18S rRNA genes and ITS regions using the conspicuous tintinnid ciliates as a case study. *ISME J.* 7 244–255. 10.1038/ismej.2012.106 23038176 PMC3554406

[B3] Banerjee S. Schlaeppi K. Mga V. D. H. (2018). Keystone taxa as drivers of microbiome structure and functioning. *Nat. Rev. Microbiol.* 16 567–576. 10.1038/s41579-018-0024-1 29789680

[B4] Benesperi R. Giuliani C. Gennai M. Lippi M. M. Guidi T. Nascimbene J. (2012). Forest plant diversity is threatened by *Robinia pseudoacacia* (black-locust) invasion. *Biodivers. Conserv.* 21 3555–3568. 10.1007/s10531-012-0380-5

[B5] Bengtsson-Palme J. Ryberg M. Hartmann M. Branco S. Wang Z. Godhe A. (2013). Improved software detection and extraction of ITS1 and ITS2 from ribosomal ITS sequences of fungi and other eukaryotes for analysis of environmental sequencing data. *Methods Ecol. Evol.* 4 914–919.

[B6] Callaway R. M. Thelen G. C. Alex R. Holben W. E. (2004). Soil biota and exotic plant invasion. *Nature* 427 731–733. 10.1038/nature02322 14973484

[B7] Cierjacks A. Kowarik I. Joshi J. Hempel S. Ristow M. Lippe M. V. D. (2013). Biological flora of the British Isles: *Robinia pseudoacacia*. *J. Ecol.* 101 1623–1640.

[B8] Cui Y. Fang L. Guo X. Han F. Zhang X. (2018). Natural grassland as the optimal pattern of vegetation restoration in arid and semi-arid regions: evidence from nutrient limitation of soil microbes. *Sci. Total Environ.* 648 388–397. 10.1016/j.scitotenv.2018.08.173 30121038

[B9] De V. F. T. Griffiths R. I. Mark B. Craig H. Girlanda M. Gweon H. S. (2018). Soil bacterial networks are less stable under drought than fungal networks. *Nat. Commun.* 9:3033. 10.1038/s41467-018-05516-7 30072764 PMC6072794

[B10] Duan L. Huang M. Li Z. Zhang Z. Zhang L. (2017). Estimation of spatial mean soil water storage using temporal stability at the hill slope scale in black locust (*Robinia pseudoacacia*) stands. *Catena* 156 51–61. 10.1016/j.catena.2017.03.023

[B11] Eklind Y. Kirchmann H. (2000). Composting and storage of organic household waste with different litter amendments. II: nitrogen turnover and losses. *Bioresour. Technol.* 74 125–133. 10.1016/s0960-8524(00)00005-5 15212913

[B12] Garbary D. J. Hill N. M. Miller A. G. (2013). Invasion of *Rosa rugosa* (*Rugosa Rose*) into coastal plant communities of Brier Island, Nova Scotia. *Can. Nat.* 127 319–331.

[B13] Guo Y. Q. Hou L. J. Zhang Z. Y. Zhang J. L. Cheng J. M. Wei G. H. (2019). Soil microbial diversity during 30 years of grassland restoration on the Loess Plateau, China: tight linkages with plant diversity. *Land Degrad. Dev.* 30 1172–1182. 10.1002/ldr.3300

[B14] Heikki S. Mary A. M. (2004). Decomposition rate of organic substrates in relation to the species diversity of soil saprophytic fungi. *Oecologia* 139 98–107. 10.1007/s00442-003-1478-y 14740289

[B15] Hiiesalu I. Bahram M. Tedersoo L. (2017). Plant species richness and productivity determine the diversity of soil fungal guilds in temperate coniferous forest and bog habitats. *Mol. Ecol.* 26 4846–4858. 10.1111/mec.14246 28734072

[B16] Hui N. Jumpponen A. Francini G. Kotze D. J. Liu X. Romantschuk M. (2017). Soil microbial communities are shaped by vegetation type and park age in cities under cold climate. *Environ. Microbiol.* 19 1281–1295. 10.1111/1462-2920.13660 28063185

[B17] Inderjit van der Putten W. H. (2010). Impacts of soil microbial communities on exotic plant invasions. *Trends Ecol. Evol.* 25 512–519. 10.1016/j.tree.2010.06.006 20638747

[B18] Jain A. Sarsaiya S. Wu Q. Lu Y. Shi J. (2019). A review of plant leaf fungal diseases and its environment speciation. *Bioengineered* 10 409–424. 10.1080/21655979.2019.1649520 31502497 PMC6779379

[B19] Kaspari M. Bujan J. Weiser M. D. Ning D. Michaletz S. T. He Z. (2017). Biogeochemistry drives diversity in the prokaryotes, fungi, and invertebrates of a Panama forest. *Ecology* 98 2019–2028. 10.1002/ecy.1895 28500769

[B20] Kleinbauer I. Dullinger S. Peterseil J. Essl F. (2010). Climate change might drive the invasive tree *Robinia pseudacacia* into nature reserves and endangered habitats. *Biol. Conserv.* 143 382–390. 10.1016/j.biocon.2009.10.024

[B21] Koivusaari P. Tejesvi M. V. Tolkkinen M. Markkola A. Mykra H. Pirttila A. M. (2019). Fungi originating from tree leaves contribute to fungal diversity of litter in streams. *Front. Microbiol.* 10:651. 10.3389/fmicb.2019.00651 31001228 PMC6454979

[B22] Lazzaro L. Mazza G. D’Errico G. Fabiani A. Giuliani C. (2017). How ecosystems change following invasion by *Robinia pseudoacacia*: insights from soil chemical properties and soil microbial, nematode, micro-arthropod and plant communities. *Sci. Total Environ.* 622 1509–1518. 10.1016/j.scitotenv.2017.10.017 29054645

[B23] Liu Y. Chen X. Liu J. Liu T. Cheng J. Wei G. (2019). Temporal and spatial succession and dynamics of soil fungal communities in restored grassland on the Loess Plateau in China. *Land Degrad. Dev.* 30 1–15.

[B24] Liu Y. Zang H. Ge T. Jing B. Guggenberger G. (2018). Intensive fertilization (N, P, K, Ca, and S) decreases organic matter decomposition in paddy soil. *Appl. Soil Ecol.* 127 51–57. 10.1016/j.apsoil.2018.02.012

[B25] Ludsin S. A. Wolfe A. D. (2001). Biological invasion theory: darwin’s contributions from the origin of species. *Bioscience* 51 780–789.

[B26] Manna M. C. Jha S. Ghosh P. K. Acharya C. L. (2003). Comparative efficacy of three epigamic earthworms under different deciduous forest litters decomposition. *Bioresour. Technol.* 88 197–206. 10.1016/s0960-8524(02)00318-8 12618041

[B27] Marschner H. (1991). Mechanisms of adaptation of plants to acid soils. *Plant Soil* 134 1–20. 10.1007/bf00010712

[B28] Messier J. McGill B. J. Lechowicz M. J. (2010). How do traits vary across ecological scales? A case for trait-based ecology. *Ecol. Lett.* 13 838–848. 10.1111/j.1461-0248.2010.01476.x 20482582

[B29] Motta R. Nola P. Berretti R. (2009). The rise and fall of the black locust (*Robinia pseudoacacia* L) in the “Siro Negri” Forest Reserve (Lombardy, Italy): lessons learned and future uncertainties. *Ann. Forest Sci.* 66 410–410. 10.1051/forest/2009012

[B30] Nascimbene J. Marini L. (2010). Oak forest exploitation and black-locust invasion caused severe shifts in epiphytic lichen communities in Northern Italy. *Sci. Total Environ.* 408 5506–5512. 10.1016/j.scitotenv.2010.07.056 20709363

[B31] Nicol G. W. Sven L. Christa S. Prosser J. I. (2010). The influence of soil pH on the diversity, abundance and transcriptional activity of ammonia oxidizing archaea and bacteria. *Environ. Microbiol.* 10 2966–2978. 10.1111/j.1462-2920.2008.01701.x 18707610

[B32] Otsuki K. Yamanaka N. Du S. (2014). *Vegetation Restoration on Loess Plateau.* Chiyoda-ku: Springer.

[B33] Peay K. G. Sperber C. V. Cardarelli E. Toju H. Vitousek P. M. (2017). Convergence and contrast in the community structure of Bacteria, Fungi and Archaea along a tropical elevation-climate gradient. *FEMS Microbiol. Ecol.* 93:fix045. 10.1093/femsec/fix045 28402397

[B34] Pfeiffer B. Fender A. C. Lasota S. Hertel D. Jungkunst H. F. Daniel R. (2013). Leaf litter is the main driver for changes in bacterial community structures in the rhizosphere of ash and beech. *Appl. Soil Ecol.* 72 150–160. 10.1016/j.apsoil.2013.06.008

[B35] Qiu L. Zhang X. Cheng J. Yin X. (2010). Effects of black locust (*Robinia pseudoacacia*) on soil properties in the loessial gully region of the Loess Plateau, China. *Plant Soil* 332 207–217. 10.1007/s11104-010-0286-5

[B36] Rice S. K. Westerman B. Federici R. (2004). Impacts of the exotic, nitrogen-fixing black locust (*Robinia pseudoacacia*) on nitrogen-cycling in a pine–oak ecosystem. *Plant Ecol.* 174 97–107. 10.1023/b:vege.0000046049.21900.5a

[B37] Richardson D. M. Rejmánek M. Richardson D. M. Carruthers J. Hui C. Impson F. A. C. (2011). Trees and shrubs as invasive alien species - a global review. *Divers. Distrib.* 17 788–809. 10.1111/j.1472-4642.2011.00782.x

[B38] Robinson C. H. (2010). Cold adaptation in arctic and Antarctic fungi. *New Phytol.* 151 341–353. 10.1046/j.1469-8137.2001.00177.x

[B39] Rooney T. P. Wiegmann S. M. Rogers D. A. Waller D. M. (2010). Biotic impoverishment and homogenization in un-fragmented forest understory communities. *Conserv. Biol.* 18 787–798. 10.1111/j.1523-1739.2004.00515.x

[B40] Sarsaiya S. Jain A. Awasthi S. K. Duan Y. M. Awasthi M. K. Shi J. S. (2019a). Microbial dynamics for lignocellulosic waste bioconversion and its importance with modern circular economy, challenges and future perspectives. *Biores. Technol.* 291:121905. 10.1016/j.biortech.2019.121905 31387838

[B41] Sarsaiya S. Shi J. Chen J. (2019b). A comprehensive review on fungal endophytes and its dynamics on *Orchidaceae* plants: current research, challenges, and future possibilities. *Bioengineered* 10 316–334. 10.1080/21655979.2019.1644854 31347943 PMC6682353

[B42] Schuster B. Diekmann M. (2003). Changes in species density along the soil pH gradient-evidence from German plant communities. *Folia Geobot.* 38 367–379. 10.1007/bf02803245

[B43] Stefanowicz A. M. Stanek M. Nobis M. Zubek S. (2017). Few effects of invasive plants *Reynoutria japonica*, *Rudbeckia laciniata* and *Solidago gigantea* on soil physical and chemical properties. *Sci. Total Environ.* 574 938–946. 10.1016/j.scitotenv.2016.09.120 27665453

[B44] Tao J. Bai T. Xiao R. Wang P. Wang F. Duryee A. M. (2018). Vertical distribution of ammonia-oxidizing microorganisms across a soil profile of the Chinese Loess Plateau and their responses to nitrogen inputs. *Sci. Total Environ.* 635 240–248. 10.1016/j.scitotenv.2018.04.104 29665543

[B45] Tateno R. Tokuchi N. Yamanaka N. Sheng D. Otsuki K. Shimamura T. (2007). Comparison of litter fall production and leaf litter decomposition between an exotic black locust plantation and an indigenous oak forest near Yan’an on the Loess Plateau, China. *Forest Ecol. Manag.* 241 84–90. 10.1016/j.foreco.2006.12.026

[B46] Toledo M. Poorter L. Pena-Claros M. Alarcon A. Balcazar J. Leano C. (2011). Climate is a stronger driver of tree and forest growth rates than soil and disturbance. *J. Ecol.* 99 254–264. 10.1111/j.1365-2745.2010.01741.x

[B47] Ussiri D. A. N. Lal R. Jacinthe P. A. (2006). Soil properties and carbon sequestration of afforested pastures in reclaimed mine soils of Ohio. *Soil Sci. Soc. Am. J.* 70 1797–1806. 10.2136/sssaj2005.0352 17965380

[B48] Vick-Majors T. J. Priscu J. C. Amaral-Zettler L. A. (2014). Modular community structure suggests metabolic plasticity during the transition to polar night in ice-covered Antarctic lakes. *ISME J.* 8:778. 10.1038/ismej.2013.190 24152712 PMC3960534

[B49] Vítková M. Tonika J. Müllerová J. (2015). Black locust-successful invader of a wide range of soil conditions. *Sci. Total Environ.* 505 315–328. 10.1016/j.scitotenv.2014.09.104 25461033

[B50] Wang Y. Shao M. A. Zhang C. Han X. Mao T. (2015). Choosing an optimal land-use pattern for restoring eco-environments in a semiarid region of the Chinese Loess Plateau. *Ecol. Eng.* 74 213–222. 10.1016/j.ecoleng.2014.10.001

[B51] Yang Y. Dou Y. An S. (2018). Testing association between soil bacterial diversity and soil carbon storage on the Loess Plateau. *Sci. Total Environ.* 626 48–58. 10.1016/j.scitotenv.2018.01.081 29335174

[B52] Yang Y. Dou Y. Huang Y. An S. (2017). Links between soil fungal diversity and plant and soil properties on the Loess Plateau. *Front. Microbiol.* 8:2198. 10.3389/fmicb.2017.02198 29163460 PMC5682006

[B53] Yüksek T. (2012). The restoration effects of black locust (*Robinia pseudoacacia* L) plantation on surface soil properties and carbon sequestration on lower hills lopes in the semi-humid region of crouch drainage basin in Turkey. *Catena* 90 18–25. 10.1016/j.catena.2011.10.001

[B54] Zhang X. Liu J. Welham C. Liu C. Li D. Chen L. (2006). The effects of clonal integration on morphological plasticity and placement of daughter ramets in black locust (*Robinia pseudoacacia*). *Flora* 201 547–554. 10.1016/j.flora.2005.12.002

[B55] Zhao M. Lu X. Zhao H. Yang Y. Hale L. Gao Q. (2019). *Ageratina adenophora* invasions are associated with microbial mediated differences in biogeochemical cycles. *Sci. Total Environ.* 677 47–56. 10.1016/j.scitotenv.2019.04.330 31051382

