# Supplementary material for: Identification of Fungal Dynamics Associated With Black Locust Leaves Mineralization and Their Correlations With Physicochemical Factors
Source: Front Microbiol. 2020 Apr 7;11:348. doi: 10.3389/fmicb.2020.00348 (PMC7154111; doi:10.3389/fmicb.2020.00348)
Supplement: Supplementary file 1 [file Data_Sheet_1.pdf]

SUPPORTING INFORMATION

Identification of fungal dynamics associated with black locust leaves mineralization and their correlations with physicochemical factors

Sihui Chen[1], Jing Zhang[23], Zhongming Wen[1*]

[1]*College of Grassland Agriculture, Northwest A&F University, Yangling, Shaanxi 712100, P. R. China*

[2]*Shaanxi Provincial Land Engineering Construction Group Co., Ltd., Xi'an 710075, China*

[3]*Institute of Land Engineering and Technology, Shaanxi Provincial Land Engineering Construction Group Co., Ltd.， Xi'an,China*

* Corresponding author: zmwen@ms.iswc.ac.cn

Table s1

Comparison of soil characteristics between different zones and vegetation. Significance codes: ‘**’ 0.01 ‘*’ 0.05

	Soil organic carbon	Total nitrogen	Total phosphorus	pH value	total
Zone	<0.01 **	<0.01 **	<0.01 **	<0.01 **	<0.01 **
vegetation	<0.01 **	<0.01 **	0.95	0.46	0.50

Note: C indicated soil organic carbon, N indicated total nitrogen, P indicated total phosphorus, pH indicated pH value.

Table s2

Temperature and humidity recorded by ibuttons every 30 minutes for a week. Results are mean of replicates ± standard deviation.

Type	TA	HA	TB	HB	TC	HC
FL	21.34±1.19ab	48.07±4.79a	20.95±1.7a	59.59±10.1ab	19.29±1.49ab	92.07±1.79a
FN	20.94±0.15ab	49.09±1.93ab	20.51±0.4ab	56.13±4ab	18.36±0.44ab	95.53±1.53bc
EL	21.9±0.61a	42.19±8.74a	22.71±0.07a	50.55±9.55a	19.97±0.77a	92.77±0.45a
EN	19.73±0.37c	58.05±5.29c	20.64±3.15b	69.78±2.8b	19.81±4.27c	80.35±3.61d
SL	20.53±0.73b	52.06±2.92bc	22.96±2.61ab	55.49±4.83b	20.77±2.1bc	89.57±3.86bc
SN	20.3±1.03bc	51.73±4.74bc	19.91±1.65b	61.61±12.27b	22.48±1.84bc	87.75±6.71cd

Note: HA-humidity at 1.5 m above the ground, HB-humidity at the soil surface, HC-humidity at -10 cm soil depth, TA-temperature at 1.5 m above the ground, TB- temperature at the soil surface, TC- temperature at -10 cm soil depth.

Table s3

Statistical results of high-throughout sequencing of the ITS sequence in soil fungal communities of different vegetation type and habitat type

Type	Raw.reads	Reads.analyzed	Number of OTU97	Number of genera	Number of order	Number of class	Number of phylum
FL	83974	83457	880	278	86	24	6
FN	81080	80487	923	285	86	24	6

EL	83438	83120	714	207	68	20	5
EN	87576	86873	928	231	83	24	6
SL	84796	84022	700	269	78	26	6
SN	86368	85724	761	298	85	26	6

Note: FL-black locust in forest habitat, FN-native plants in forest habitat, EL-black locust in forest-steppe habitat, EN-native plants in forest-steppe habitat, SL-black locust in steppe habitat, SN-native plants in steppe habitat. Same is as follows.

Table s4

Statistical analysis of soil fungal community among groups contributing to their variation with three different approaches, and based on Bray-Curtis distances

Group	MRPP		Anoism		Adonis	
	δ	P	R	P	R2	P
SL-SN	0.06472	**0.001**	0.5213	**0.001**	0.16597	**0.001**
EL-EN	0.1327	0.026	0.3646	0.064	0.20894	**0.001**
FL-FN	0.1585	**0.001**	0.8848	**0.001**	0.33939	**0.001**

Note: FL-black locust in forest habitat, FN-native plants in forest habitat, EL-black locust in forest-steppe habitat, EN-native plants in forest-steppe habitat, SL-black locust in steppe habitat, SN-native plants in steppe habitat. Significant analysis based on 999 times permutation test; bold p values indicate significant difference ($p < 0.05$)

Table s5

Topological features of the co-occurrence networks of soil fungal communities in the grassland restored for different durations

Vegetation type	Node	Edge	Average path length	Clustering coefficient	Modularity index
FL	636	2881	6.74(19)	0.305	0.757
FN	705	4216	5.92(20)	0.426	0.715
SL	668	3847	4.68(11)	0.379	0.672
SN	759	6675	4.45(11)	0.416	0.619

Note: FL-black locust in forest habitat, FN-native plants in forest habitat, EL-black locust in forest-steppe habitat, EN-native plants in forest-steppe habitat, SL-black locust in steppe habitat, SN-native plants in steppe habitat.

Table s6

Betweenness centrality values of keystone species (genus) in the co-occurrence networks of soil fungal communities

Type	Genus	Betweenness centrality
FN	Cladophialophora	308.0
	Geastrum	280.0
	Hygrocybe	274.0
	Beauveria	273.5
	Hirsutella	243.0
SN	Stanjemonium	405.2
	Aspergillus	383.7
	Eremiomyces	376.2
	Scolecobasidium	320.5
	Zopfiella	252.9

Note: FN-native plants in forest habitat, SN-native plants in steppe habitat.

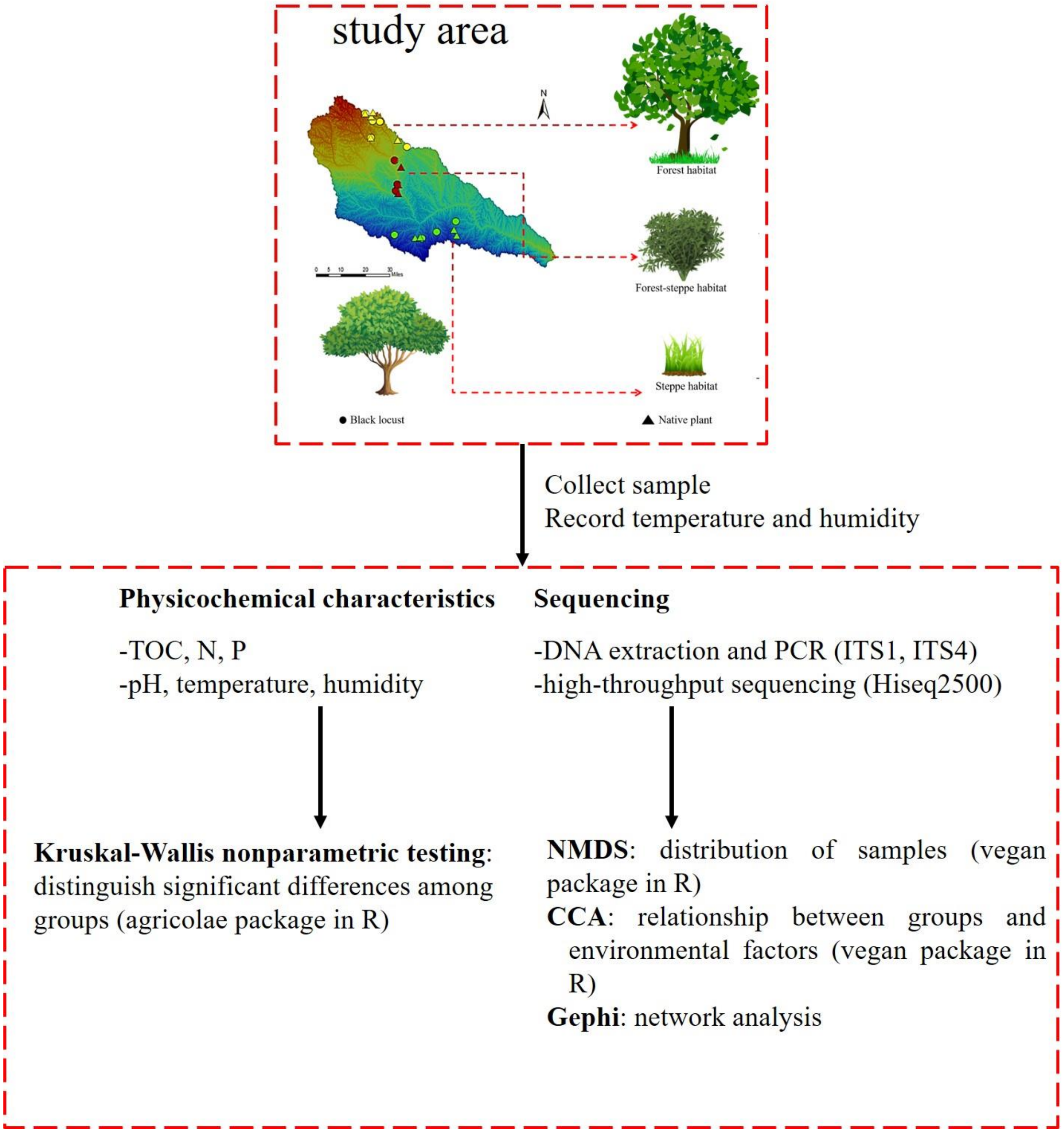

Figs. 1. Methods followed in this study were provided in a schematic flow diagram for better understanding. FL-black locust in forest habitat, FN-native plants in forest habitat, EL-black locust in forest-steppe habitat, EN-native plants in forest-steppe habitat, SL-black locust in steppe habitat, SN-native plants in steppe habitat, TOC-total organic carbon, N-total nitrogen, P-total phosphorus, NMDS-NonMetric MultiDimensional Scaling, CCA-Canonical correspondence analysis.

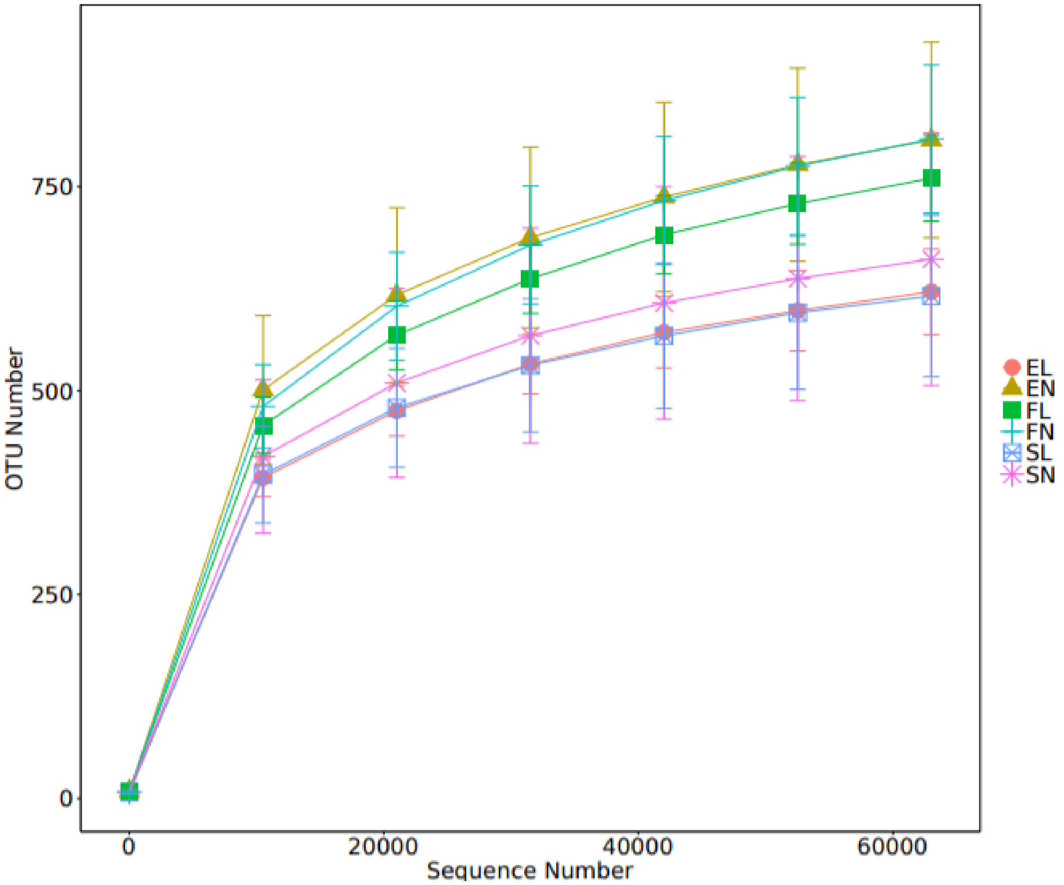

Figs. 2. Rarefaction curves of six group, includes native plant and black locust in forest, forest-steppe, steppe habitat, separately. FL-black locust in forest habitat, FN-native plants in forest habitat, EL-black locust in forest-steppe habitat, EN-native plants in forest-steppe habitat, SL-black locust in steppe habitat, SN-native plants in steppe habitat.

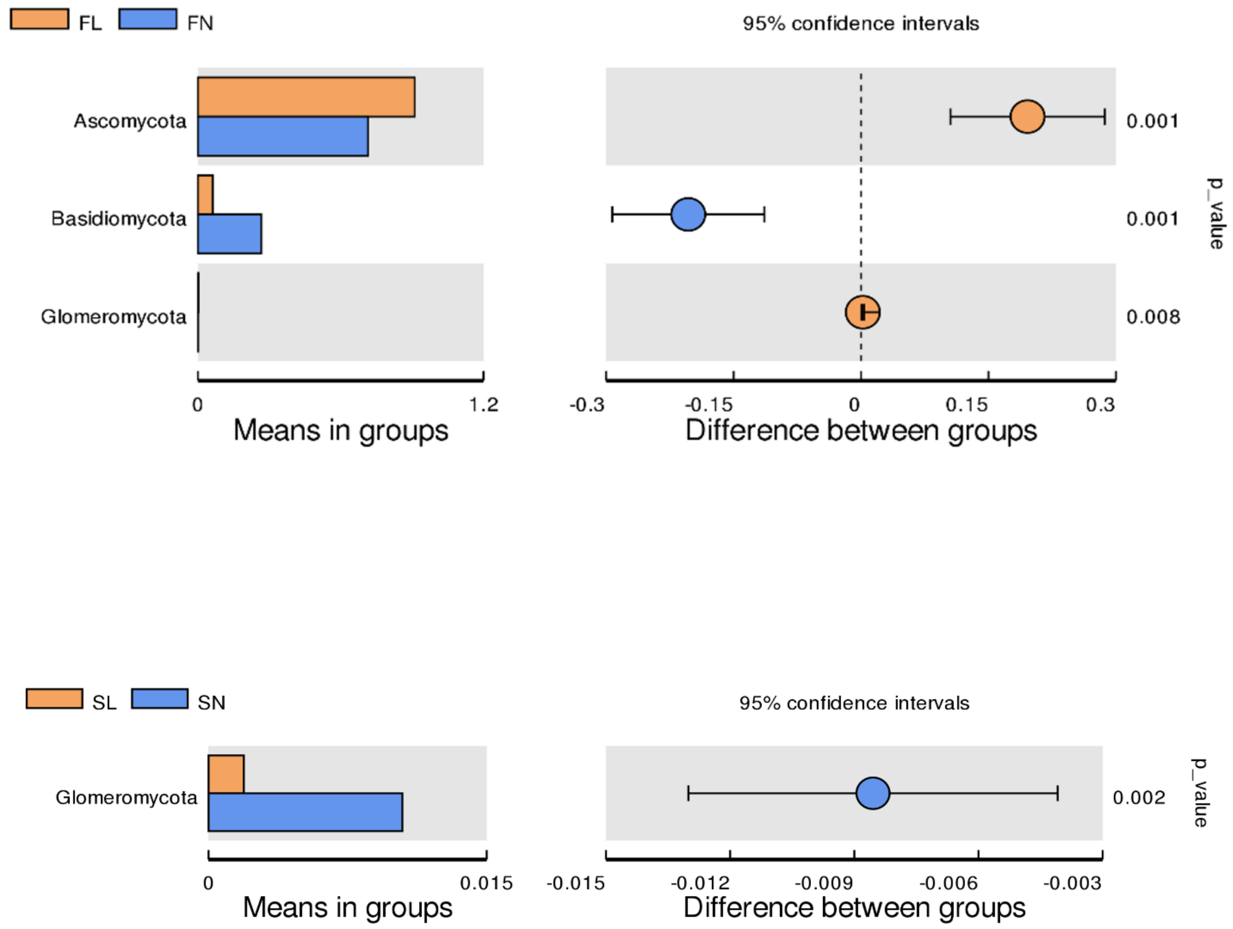

Figs. 3. Composition difference of phylum between invasion plant group and native plant group. T test was used to determine the difference significance (*P* <0.05). The invasion of Black locust increased the relative abundances of *Ascomycota*, *Glomeromycota* in forest zone and decreased the *Basidiomycota* in forest zone and *Glomeromycota* in steppe zone. FL-black locust in forest habitat, FN-native plants in forest habitat, SL-black locust in steppe habitat, SN-native plants in steppe habitat.

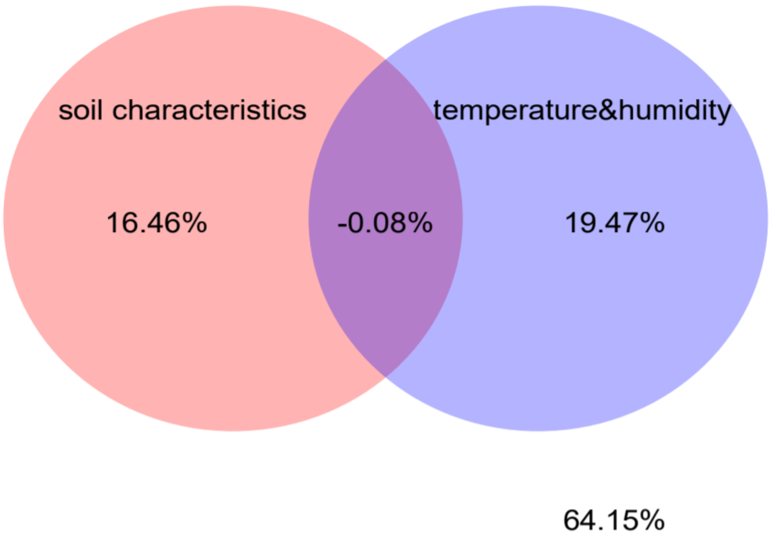

Figs. 4. The Variance partitioning canonical correspondence analysis (VPA) shows the factors that affected the fungi composition and proportion.

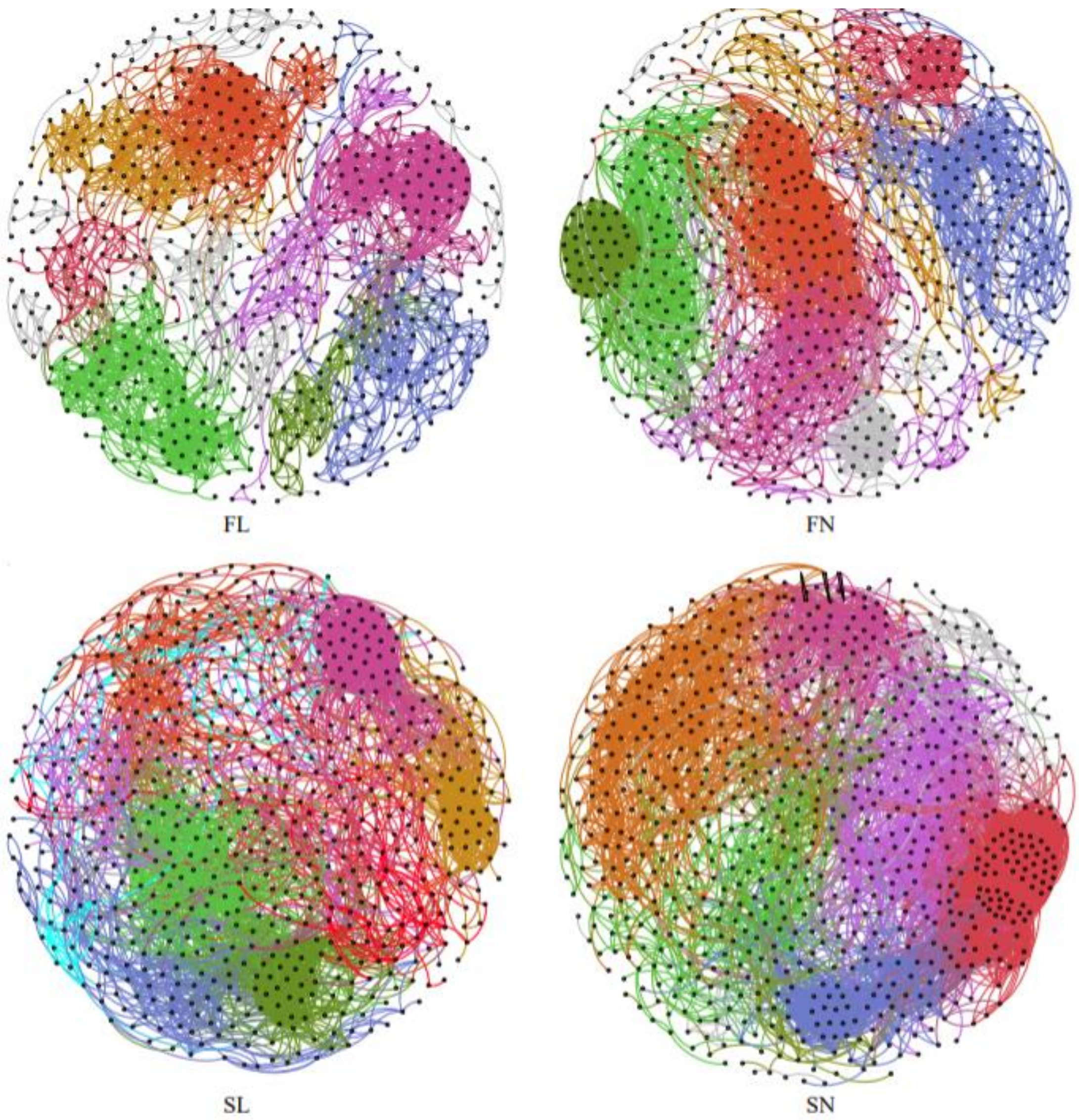

Figs. 5. Modular structure network of fungi, (a) black locust in the forest habitat, (b) native plants in the forest habitat, (c) black locust in the steppe habitat, (d) native plants in the steppe habitat. FL-black locust in forest habitat, FN-native plants in forest habitat, SL-black locust in steppe habitat, SN-native plants in steppe habitat.
